# A new species of *Pholcusyichengicus* species-group (Araneae, Pholcidae) from Hebei Province, China

**DOI:** 10.3897/BDJ.10.e81800

**Published:** 2022-02-21

**Authors:** Ying Lu, Zhiyuan Yao, Qiaoqiao He

**Affiliations:** 1 College of Life Science, Shenyang Normal University, Shenyang, China College of Life Science, Shenyang Normal University Shenyang China

**Keywords:** taxonomy, morphology, biodiversity, daddy-long-legs spider

## Abstract

**Background:**

The *Pholcusyichengicus* species-group currently contains 37 species. It is distributed in central and south-eastern China and Thailand, except for *P.guani* Song & Ren, 1994 from Liaoning Province, north-eastern China and *P.clavatus* Schenkel, 1936 which is widely distributed in the country.

**New information:**

*Pholcusbajia* sp. nov. is described as a new species of the *P.yichengicus* species-group collected from Hebei Province, China.

## Introduction

**Figure 1.**
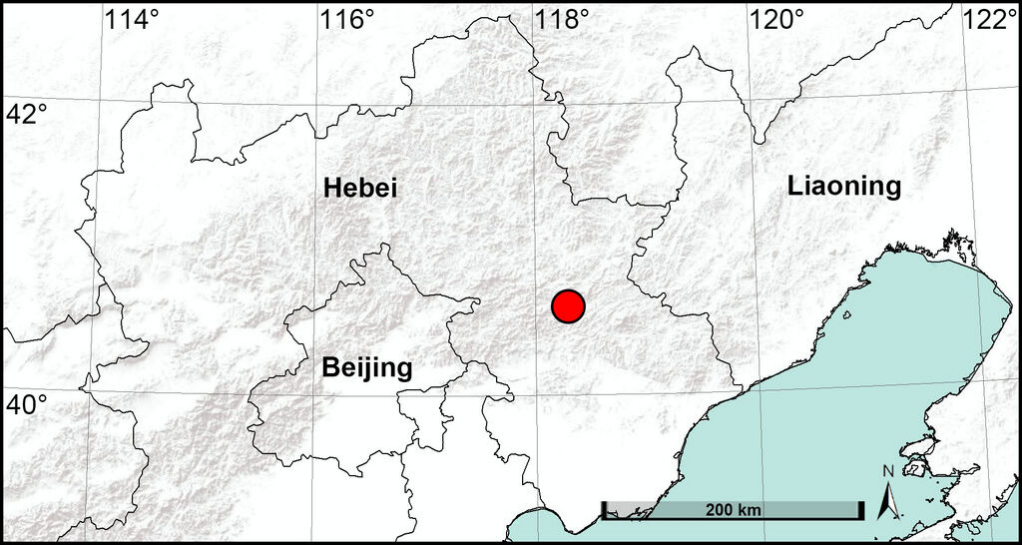
Distribution record of *Pholcusbajia* sp. nov. in Hebei Province, China.

Pholcidae C.L. Koch, 1850 contains 96 genera and 1850 species (World Spider Catalog 2022). *Pholcus* Walckenaer, 1805 is the largest pholcid genus that currently contains 361 species belonging to 21 species-groups (Huber 2011, Lan et al. 2020, World Spider Catalog 2022). The *P.yichengicus* species-group contains 37 species (Huber 2011,Dong et al. 2016a, Dong et al. 2016b, Dong et al. 2017, Zhu et al. 2018, Lan et al. 2020), 31 of these being found in central and south-eastern China; *P.guani* Song & Ren, 1994 is known from Liaoning Province, north-eastern China and *P.clavatus* Schenkel, 1936 is the only species with a wide distribution in the country. In this paper, we describe a new species of the *P.yichengicus* species-group, based on material of both sexes collected in Hebei Province, China (Fig. 1).

## Materials and methods

Specimens were examined and measured with a Leica M205 C stereomicroscope. The left male pedipalp was photographed. External female genitalia was photographed before the dissection. Vulva was treated in a 10% warm solution of potassium hydroxide (KOH) to dissolve soft tissues before illustration. Images were captured with a Canon EOS 750D wide zoom digital camera (24.2 megapixels) mounted on the stereomicroscope mentioned above and assembled using Helicon Focus 3.10.3 image stacking software (Khmelik et al. 2005). All measurements are given in millimetres (mm). Leg measurements are shown as: total length (femur, patella, tibia, metatarsus, tarsus). Leg podomeres were measured on their dorsal side. The distribution map was generated with ArcGIS 10.2 (Esri Inc.). The specimens studied are preserved in 75% ethanol and deposited in the College of Life Science, Shenyang Normal University (SYNU) in Liaoning, China. Terminology and taxonomic descriptions follow Huber (2011), Yao et al. (2015) and Yao et al. (2021). The following abbreviations are used in the descriptions: ALE = anterior lateral eye, AME = anterior median eye, PME = posterior median eye, L/d = length/diameter; used in the illustrations: a = appendix, b = bulbal, da = distal apophysis, e = embolus, fa = frontal apophysis, pa = proximo-lateral apophysis, pp = pore plate, pr = procursus and u = uncus.

## Taxon treatments

### Pholcus bajia sp. n.

20EAAAAE-DCEF-59AD-B874-A0538B5299EB

FF35AB52-DBFC-4F2D-BFF8-33DA23A8EF69

#### Materials

- **Type status:** Holotype. **Occurrence:** recordedBy: Zhiyuan Yao, Ying Lu and Fangyu Zhao; individualCount: 1; sex: male; lifeStage: adult; **Taxon:** order: Araneae; family: Pholcidae; genus: Pholcus; **Location:** country: China; stateProvince: Hebei; municipality: Chengde; locality: Bajia Town; verbatimLocality: roadside of S358; verbatimElevation: 447 m a.s.l.; verbatimLatitude: 40°38.46'N; verbatimLongitude: 118°17.63'E; **Event:** samplingProtocol: by hand; year: 2021; month: 7; day: 28; **Record Level:** institutionCode: SYNU-Ar00247
- **Type status:** Paratype. **Occurrence:** recordedBy: Zhiyuan Yao, Ying Lu and Fangyu Zhao; individualCount: 3; sex: 1 male, 2 females; lifeStage: adult; **Taxon:** order: Araneae; family: Pholcidae; genus: Pholcus; **Location:** country: China; stateProvince: Hebei; municipality: Chengde; locality: Bajia Town; verbatimLocality: roadside of S358; verbatimElevation: 447 m a.s.l.; verbatimLatitude: 40°38.46'N; verbatimLongitude: 118°17.63'E; **Event:** samplingProtocol: by hand; year: 2021; month: 7; day: 28; **Record Level:** institutionCode: SYNU-Ar00248–00250

#### Description

**Figure 2.**
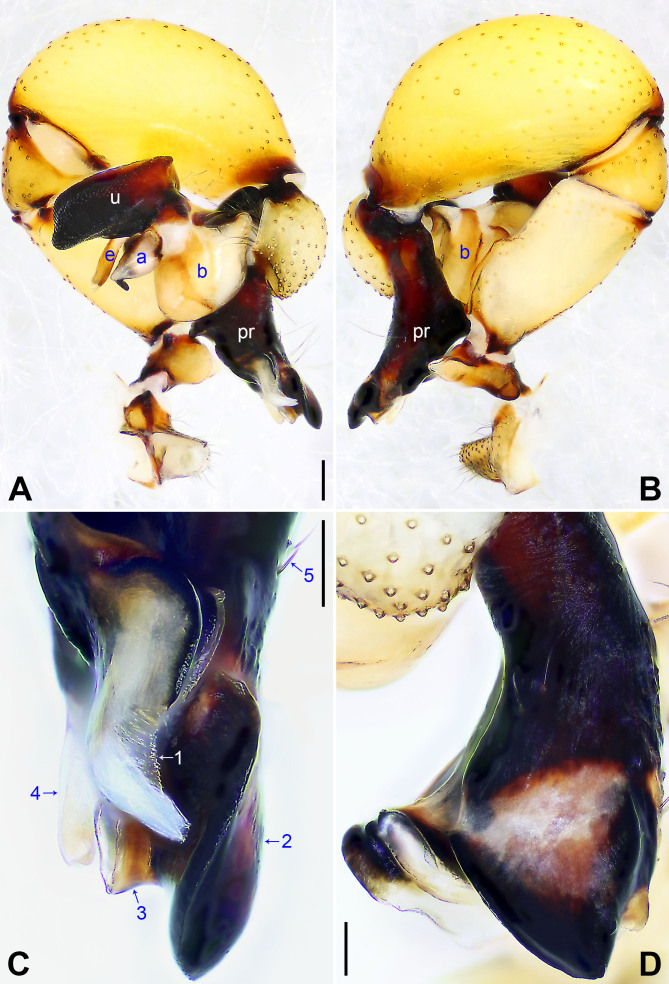
*Pholcusbajia* sp. nov., holotype male. **A** Pedipalp, prolateral view; **B** Pedipalp, retrolateral view; **C** Distal part of procursus, prolateral view, arrows 1–5 point at prolateral membranous process, prolatero-dorsal apophysis, distal apophysis, ventral membranous process and dorsal spines, respectively; **D** Distal part of procursus, dorsal view. a = appendix, b = bulb, e = embolus, pr = procursus, u = uncus. Scale bars: 0.20 mm (A–B), 0.10 mm (C–D).

**Figure 3.**
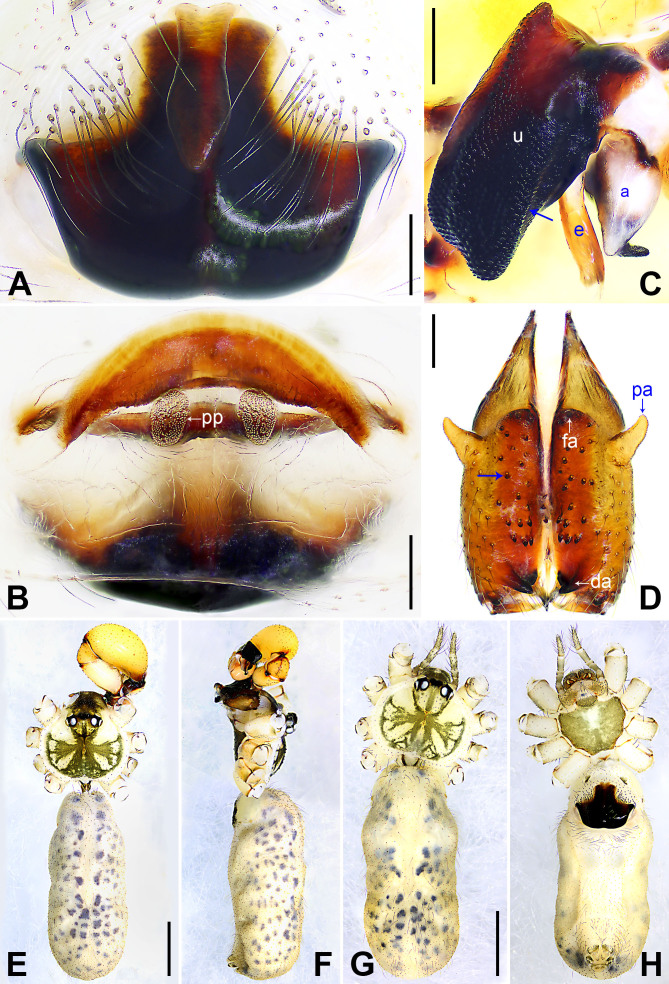
*Pholcusbajia* sp. nov. **A** Paratype female, external genitalia, ventral view; **B** Paratype female, vulva, dorsal view; **C** Holotype male, bulbal apophyses, prolateral view, arrow points at median protrusion; **D** Holotype male, chelicerae, frontal view, arrow points at median cones; **E** Holotype male, habitus, dorsal view; **F** Holotype male, habitus, lateral view; **G** Paratype female, habitus, dorsal view; **H** Paratype female, habitus, ventral view. a = appendix, da = distal apophysis, e = embolus, fa = frontal apophysis, pa = proximo-lateral apophysis, pp = pore plate, u = uncus. Scale bars: 0.20 mm (A–D), 1.00 mm (E–H).

**Male** (holotype): Total length 5.31 (5.54 with clypeus), carapace 1.31 long, 1.87 wide, opisthosoma 4.00 long, 1.70 wide. Leg I: 48.10 (12.16, 0.76, 11.98, 20.56, 2.64), leg II missing, leg III: 22.32 (6.56, 0.67, 5.28, 8.68, 1.13), leg IV: 30.61 (8.89, 0.69, 7.59, 12.02, 1.42); tibia I L/d: 71. Eye sizes and their interdistances: PME 0.19, PME–PME 0.29, PME–ALE 0.04, AME 0.13, AME–AME 0.08. Sternum wider than long (1.28/0.95). Habitus as in Fig. 3E–F. Carapace yellowish, with brown radiating marks and marginal brown bands; ocular area yellowish, with median and lateral brown bands; clypeus yellowish, with brown marks; sternum dark brown. Legs yellowish, but dark brown on patellae and whitish on distal parts of femora and tibiae, with darker rings on subdistal parts of femora and proximal and subdistal parts of tibiae. Opisthosoma yellowish, with dorsal and lateral spots. Chelicerae (Fig. 3D) with pair of proximo-lateral apophyses, pair of distal apophyses without teeth, pair of frontal apophyses and several small median cones (arrowed in Fig. 3D). Pedipalp as in Fig. 2A–B; trochanter with long (longer than wide), retrolaterally strongly bulged ventral apophysis; femur with small retrolatero-proximal apophysis and indistinct ventral protuberance; tibia with prolatero-ventral projection; procursus simple proximally, but complex distally, with curved, prolateral membranous process (arrowed 1 in Fig. 2C), sclerotised prolatero-dorsal apophysis (arrowed 2 in Fig. 2C), slightly sclerotised distal apophysis (arrowed 3 in Fig. 2C), ventral membranous process (arrowed 4 in Fig. 2C) and three slender dorsal spines (arrowed 5 in Fig. 2C); uncus with scales, medially protruding (arrowed in Fig. 3C); appendix hooked, with subdistal membranous branch (Fig. 3C); embolus weakly sclerotised, with some transparent distal projections (Fig. 3C). Retrolateral trichobothrium of tibia I at 5% proximally; legs with short vertical setae on tibiae, metatarsi and tarsi, without spines or curved setae; tarsus I with 27 distinct pseudo-segments.

**Female**: Similar to male, habitus as in Fig. 3G–H. Total length 4.53 (4.74 with clypeus), carapace 1.25 long, 1.60 wide, opisthosoma 3.28 long, 1.59 wide; tibia I: 7.40; tibia I L/d: 49. Eyes sizes and their interdistances: PME 0.14, PME–PME 0.21, PME–ALE 0.05, AME 0.09, AME–AME 0.04. Sternum wider than long (1.03/0.75). Sternum yellowish, with brown marks. External female genitalia (Fig. 3A) dark brown, slightly curved postero-medially, with wedge-shaped knob. Vulva (Fig. 3B) with crescent-shaped, sclerotised anterior arch and pair of nearly elliptic pore plates.

**Variation**: Tibia I in the paratype male (SYNU-Ar00248): 11.20. Tibia I in a paratype female (SYNU-Ar00250): 8.20.

#### Diagnosis

The species resembles *P.harveyi* Zhang & Zhu, 2009 (see Zhang and Zhu 2009: fig. 15; Yao and Li 2012: figs. 75–76) by having similar male cheliceral apophyses (Fig. 3D), but can be easily distinguished by its procursus with curved, prolateral membranous process (arrowed 1 in Fig. 2C; absent in *P.harveyi*) and ventral membranous process (arrowed 4 in Fig. 2C; ventral sclerotised apophysis in *P.harveyi*), by uncus medially protruding (arrowed in Fig. 3C; not protruding in *P.harveyi*), by appendix with subdistal membranous branch (Fig. 3C; absent in *P.harveyi*), by external female genitalia nearly anchor-shaped (Fig. 3A; nearly triangular in *P.harveyi*) and by vulval anterior arch crescent-shaped (Fig. 3B; nearly eyebrow-shaped in *P.harveyi*).

#### Etymology

The specific name refers to the type locality and is a noun in apposition.

#### Distribution

China (Hebei, type locality; Fig. 1).

#### Biology

The species was found on rock walls.

## Supplementary Material

XML Treatment for Pholcus bajia
